# Supplementary material for: Selective plasticity of fast and slow excitatory synapses on somatostatin interneurons in adult visual cortex
Source: Nat Commun. 2023 Nov 7;14:7165. doi: 10.1038/s41467-023-42968-y (PMC10630508; doi:10.1038/s41467-023-42968-y)
Supplement: Supplementary file 1 — Supplementary Information [file 41467_2023_42968_MOESM1_ESM.pdf]

Supplementary Figures

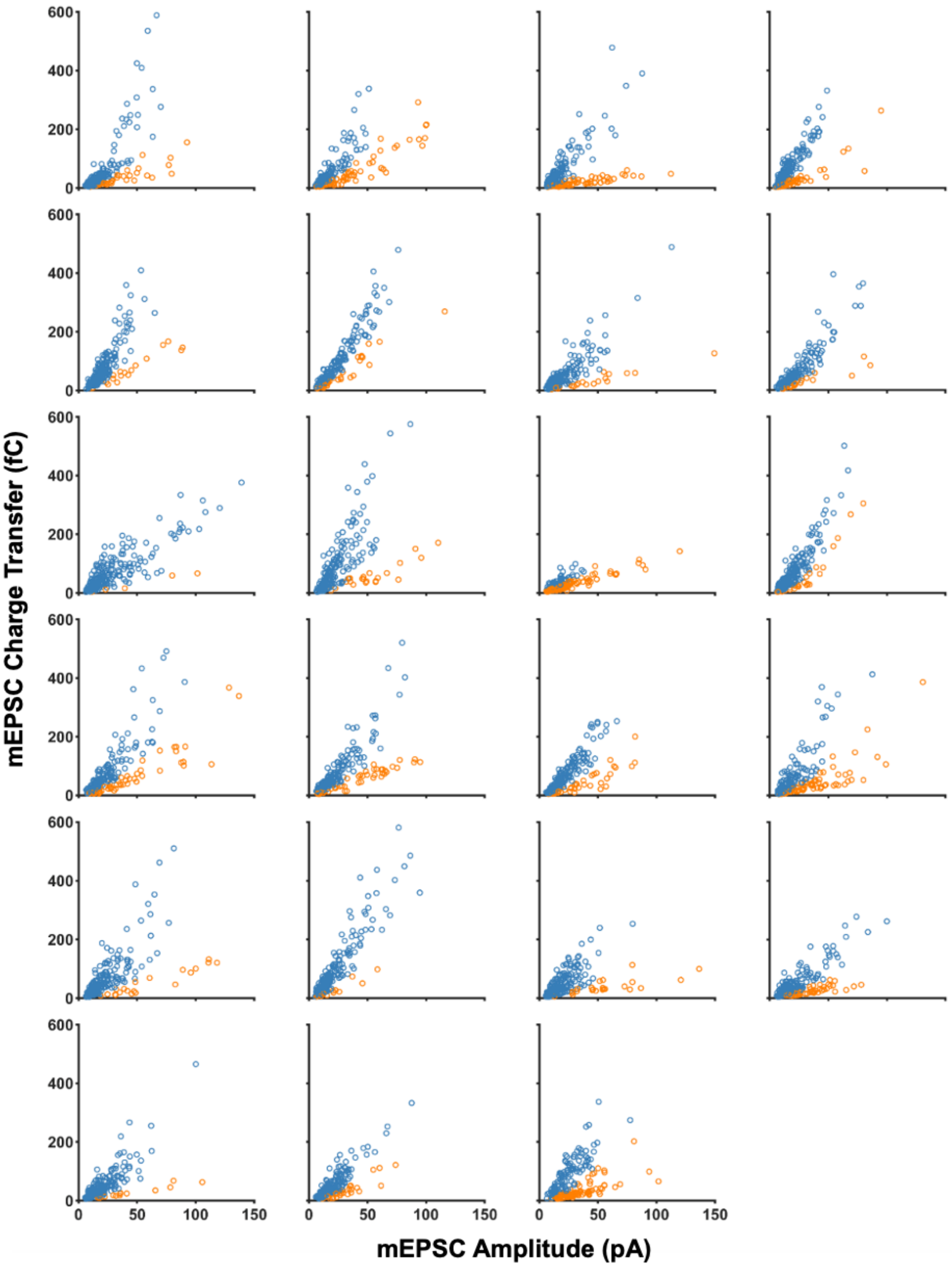

Figure S1. Two distinct types of mEPSCs in each SOM.

Each panel corresponds to a single NR cell and shows 200 mEPSCs clustered into S-Type (blue circles) and F-Type (orange circles).

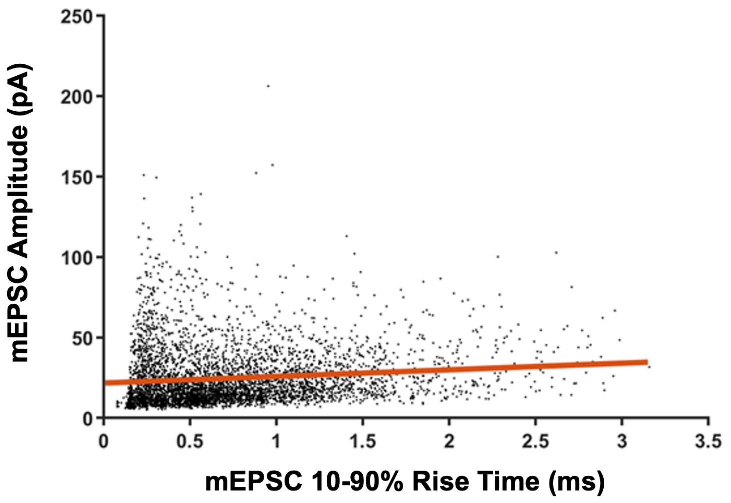

Figure S2. S-Type events are unlikely to be filtered F-Type events.

Plot of mEPSC amplitude against mEPSC 10-90% rise time for mEPSCs recorded from NR SOM cells ($n = 23$ cells, 200 events per cell). There is no negative correlation between the two parameters, which suggests that the mEPSCs are not subject to dendritic filtering (Linear regression: slope = 4.1084, ****$p < 0.0001$).

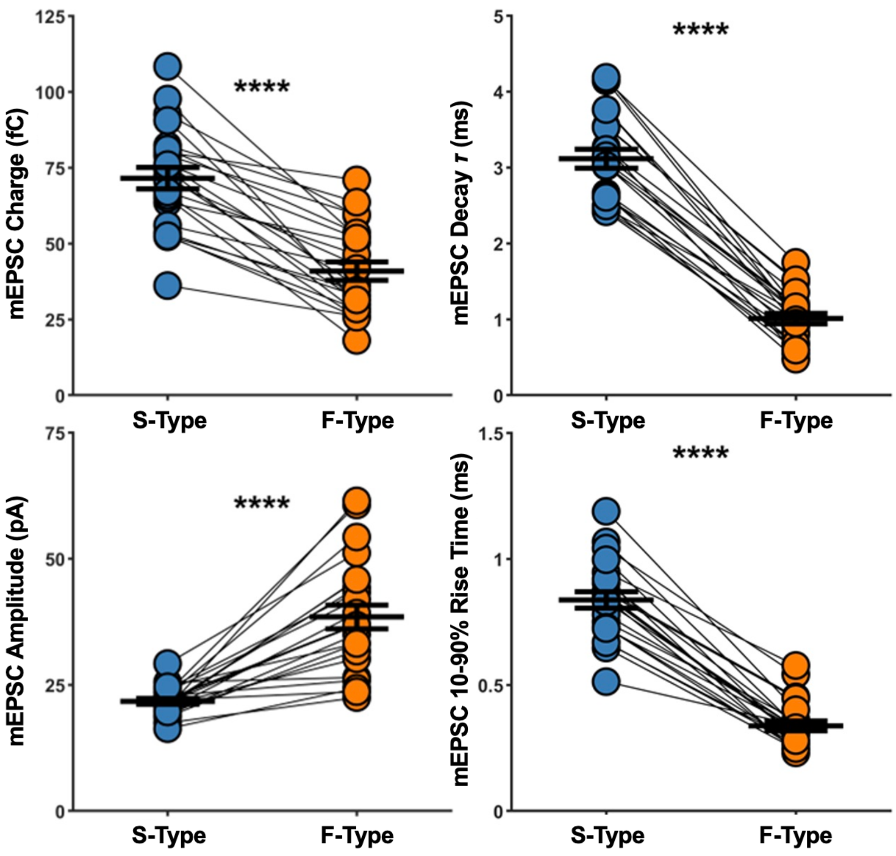

Figure S3. The two types of mEPSCs have distinct kinetics.

Data points are averages for individual cells. Lines connect the averages of S-Type and F-Type mEPSCs recorded from the same SOM. Data displayed as mean±SEM. Top left: S-Type mEPSCs have a significantly greater charge transfer (Student's paired-sample t-test, $t = 9.00$, ****$p < 0.0001$). Bottom left: F-Type mEPSCs have a significantly greater amplitude (Student's paired-sample t-test, $t = -7.57$, ****$p < 0.0001$). Top right: S-Type mEPSCs have a significantly longer decay τ (Student's paired-sample t-test, $t = 21.63$, ****$p < 0.0001$). Bottom right: S-Type mEPSCs have a significantly longer rise time (Student's paired-sample t-test, $t = 16.98$, ****$p < 0.0001$). Circles: average value for each cell. Thick black lines: group mean ± S.E.M. Thin dark gray lines connect data from the same cell.

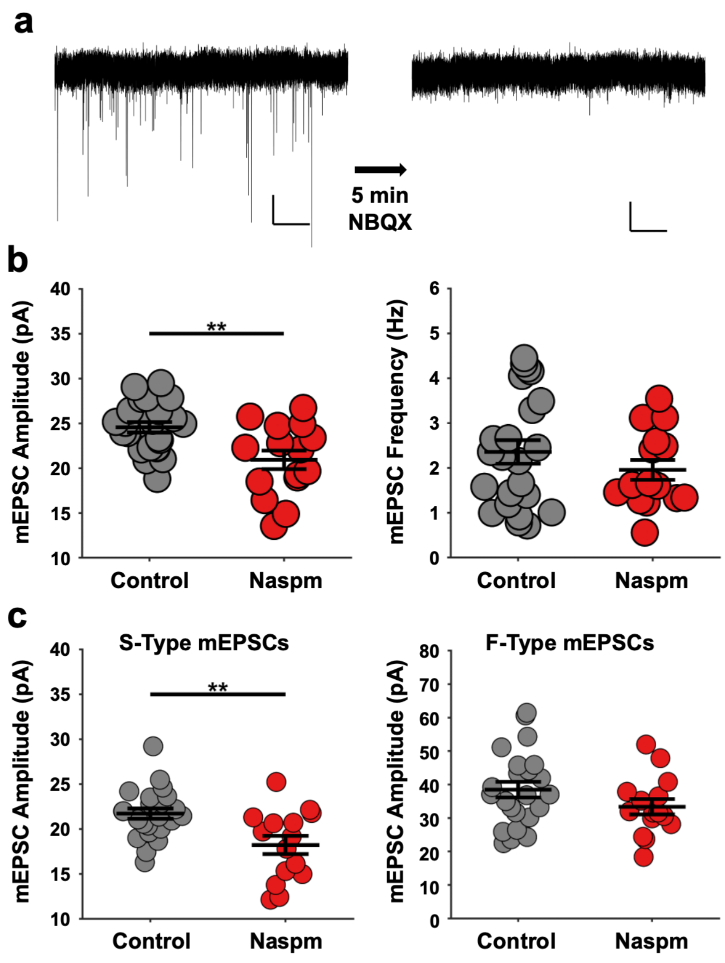

Figure S4. mEPSCs recorded from V1 L2/3 SOMs are mediated by AMPA receptors.

a. Both S-Type and F-Type synaptic currents are mediated by AMPARs. An example mEPSC trace before (left) and 5 min after bath application of 10 μM NBQX (right). Scale bars: 10 pA, 2.5 s.
b. CP-AMPARs are present at V1 L2/3 SOM synapses. Left: Comparison of average mEPSC amplitude from NR SOMs under control condition (NR) or in the presence of 20 μM Naspm (Student's two-sample t-test: $t = 3.22$, $^{**}p=0.0027$). Right: Comparison of average mEPSC frequency with or without Naspm (Student's two-sample t-test: $t = 0.95$, $p=0.3469$). Naspm and control recordings were done in different cells to keep the duration of recordings similar to avoid potential washout from prolonged whole-cell recordings.
c. Naspm significantly decreases the mEPSC amplitude of S-Type (left panel, $^{**}p<0.01$), but not F-Type (right panel). Circles: average value for each cell. Thick black lines: group mean ± S.E.M.

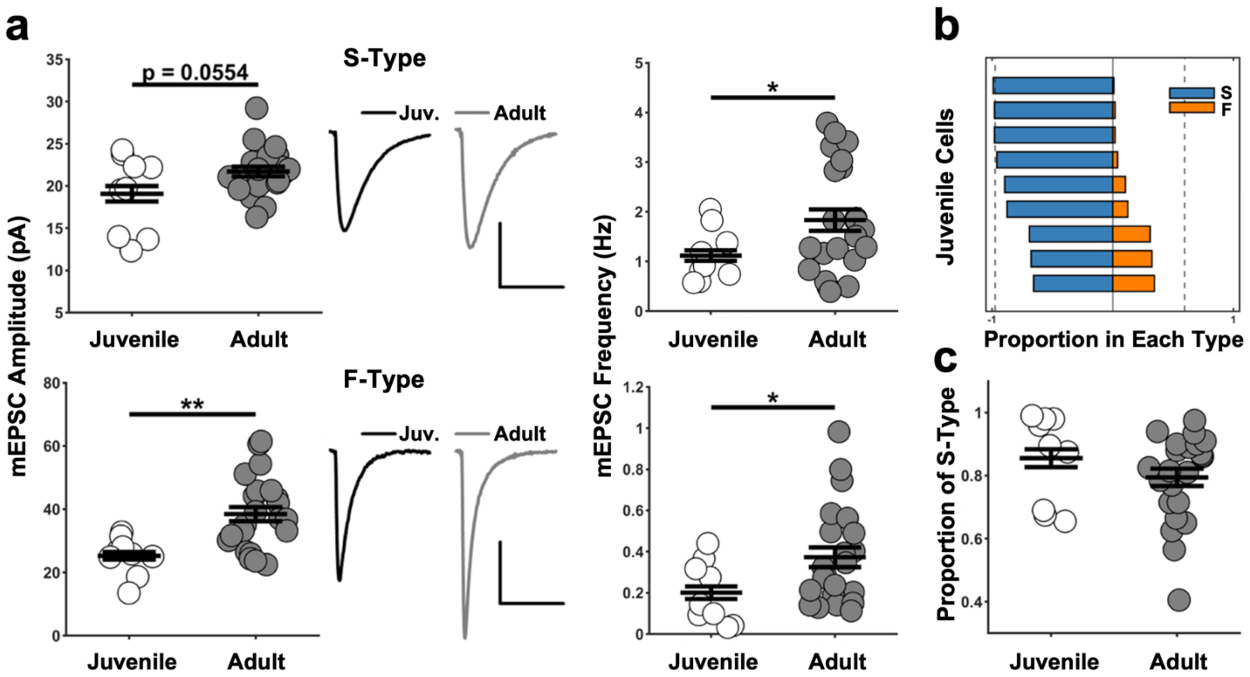

Figure S5. The two types of mEPSCs are present in juvenile V1 L2/3 SOMs and are developmentally regulated.

a. There is developmental regulation of both mEPSC types. Top left: There is a trend towards an increase in the amplitude of S-Type events (Student's two-sample t-test: t = -1.99, p = 0.0554). Top center: average S-Type mEPSCs (scale bar: 10 pA, 10 ms). Top right: There is a significant increase in the frequency of S-Type events (Student's two-sample t-test: t = -2.49, *p = 0.0189). Bottom left: There is a significant increase in the amplitude of F-Type events (Student's two-sample t-test: t = -3.33, **p = 0.0023). Bottom center: average F-Type mEPSCs (scale bar: 10 pA, 10 ms). Bottom right: There is a significant increase in the frequency of F-Type events (Student's two-sample t-test: t = -2.40, *p = 0.0247).
b. The proportion of S-Type and F-Type mEPSCs in each juvenile SOM cell. Each bar represents a cell. Dotted lines represent the bounds of proportions in adult NR SOM cells.
c. The proportion of each type across the cell population does not significantly change with development (Student's two-sample t-test: t = 1.11, p = 0.2751).

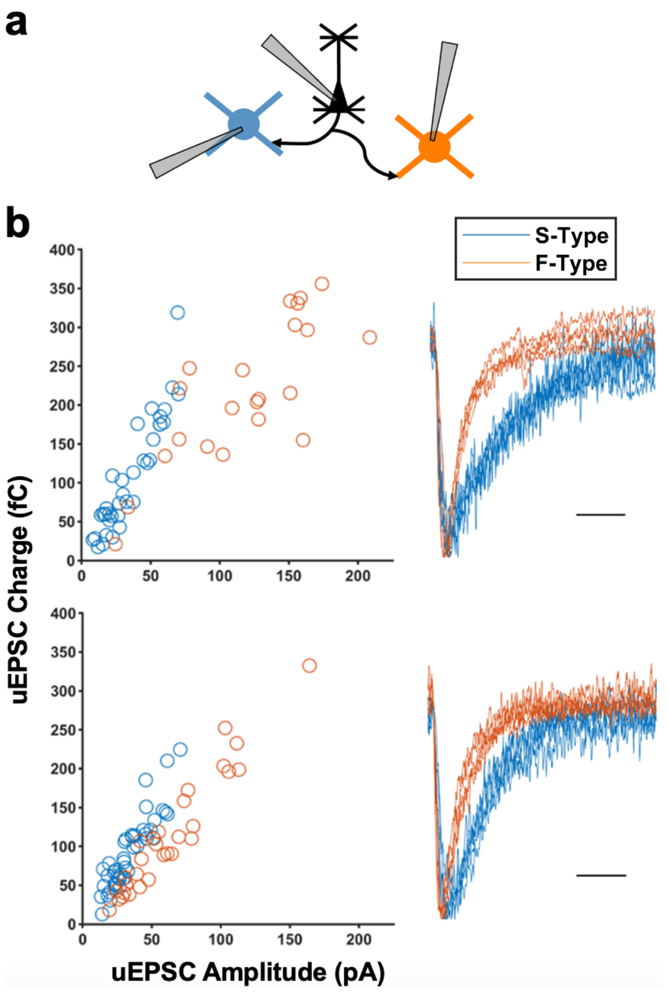

Figure S6. Connections between a single presynaptic PC and two postsynaptic V1 L2/3 SOMs show different kinetics.

a. Schematic of simultaneous recordings of one presynaptic PC and two postsynaptic SOMs
b. Example cells from Allen Institute Synaptic Physiology dataset. Each row of panels represents a separate experiment. Left: mEPSC charge transfer plotted against mEPSC amplitude for two postsynaptic SOMs. Right: randomly selected uEPSCs from each experiment. Colors correspond to colors in left panels. Amplitudes are normalized to peak. Scale bars: 10 ms.

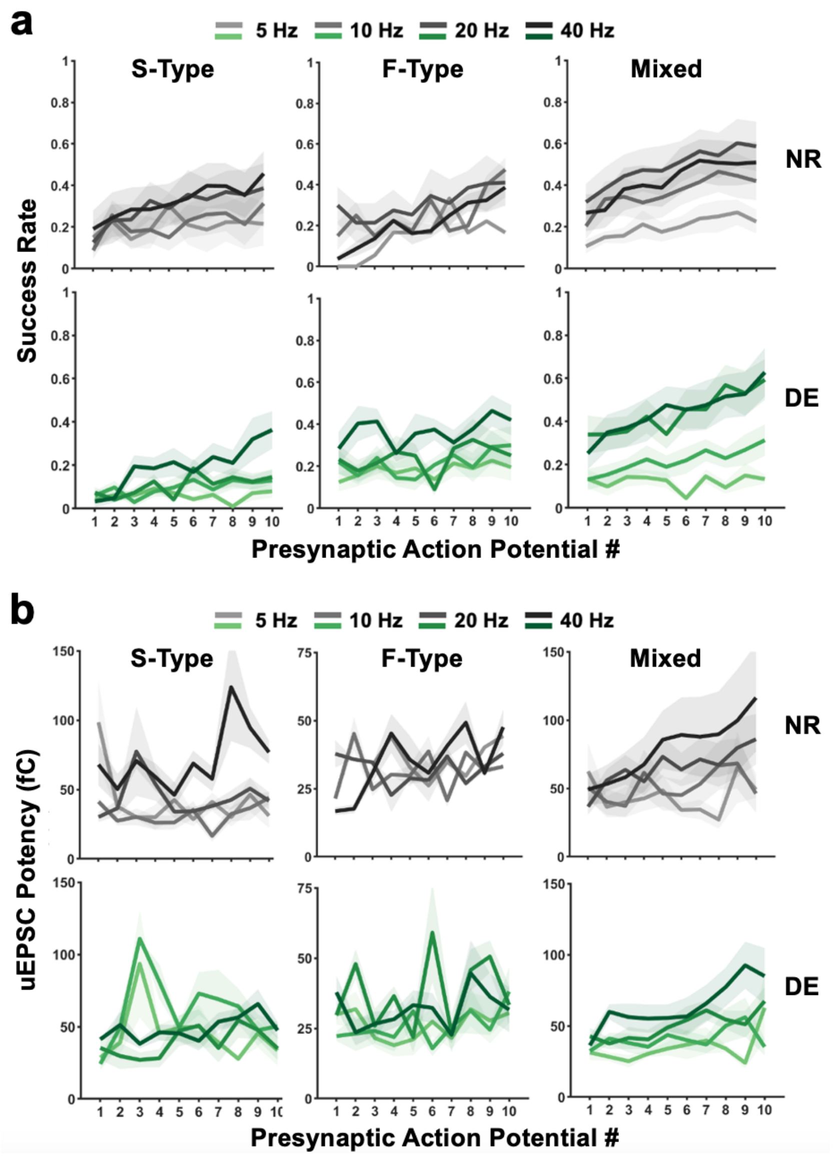

Figure S7. Comparison of success rate and potency of PC to SOM uEPSCs.

a. Comparison of uEPSC success rate for each presynaptic action potential in a train across different stimulation frequencies (5 Hz, 10 Hz, 20 Hz, and 40 Hz). Left panels: S-Type pairs. Middle panels: F-Type pairs. Right panels: Mixed pairs. Top row: Results from NR (gray). Bottom row: Results from DE (green). Lines: means values. Shaded areas: S.E.M.

b. Comparison of uEPSC potency for each presynaptic action potential in a train across different stimulation frequencies (5 Hz, 10 Hz, 20 Hz, and 40 Hz). Left panels: S-Type pairs. Middle panels: F-Type pairs. Right panels: Mixed pairs. Top row: Results from NR (gray). Bottom row: Results from DE (green). Lines: mean values. Shaded areas: S.E.M.

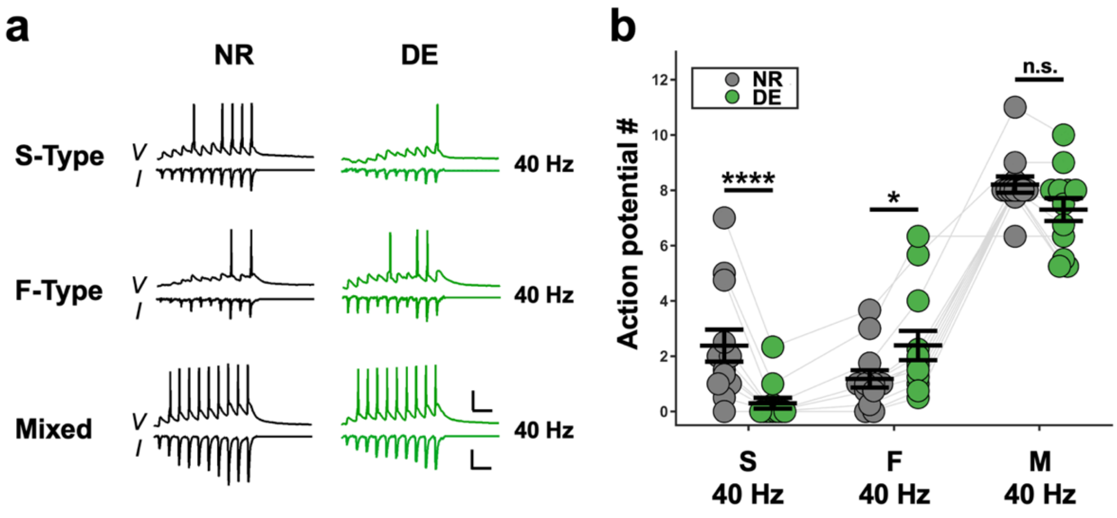

Figure S8. Playback of average uEPSC traces to SOM.

a. Average voltage (*V*) traces recorded in current-clamp playback of average uEPSC current traces (*I*) (data in Fig. 3). All traces displayed are from the same control V1 L2/3 SOM cell. Top panels: Recordings from playback of average S-Type unitary connection current traces. Middle panels: Recordings from average F-Type unitary connection current traces. Bottom panels: Recordings from average Mixed connection current traces. Left column: Playback of average current traces from NR mice (black). Right column: Playback of average current traces from DE mice (green). Scale bars: *V* traces 25 mV, 50 ms; *I* traces 250 pA, 50 ms.

b. Comparison of number of action potentials generated in SOMs while playing back the different average current traces in the presence of synaptic receptor blockers (NBQX + APV + gabazine). All SOMs that were recorded from (n = 12 cells from 4 mice) received playback of NR and DE traces for each connection type (S-Type, F-Type, and Mixed) for a total of 6 unique conditions. Circles: average for each cell. Thick black lines: group mean ± S.E.M. Gray lines connect data points obtained from the same SOM cell. Data were fit with a linear mixed effects model. Analysis of main effects and interactions can be found in Table S3. Further pairwise contrasts of estimated marginal means are shown in Table S5. A subset of these pairwise contrasts (instances of NR versus DE) is indicated above the groups (****: $p < 0.0001$, *: $p < 0.05$, n.s.: not significant).

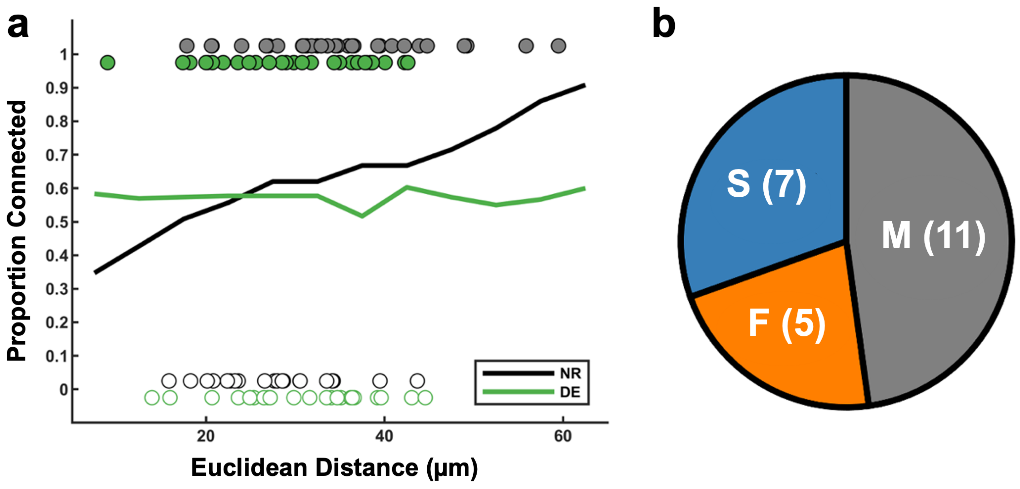

Figure S9. Changes in local connectivity following DE.

a. The observed pattern of connectivity between V1 L2/3 PCs and SOMs was influenced by both the intersomatic (Euclidean) distance between pairs and by the recent history of sensory experience, and the influence of distance varied between NR and DE (Logistic Regression; effect of distance: t = 2.61, **p = 0.0091; effect of DE: t = 2.23, *p = 0.0261; interaction between distance and DE: t = -2.28, *p = 0.0228). Lines show moving means of binary connectivity values (for display purposes only). Individual cell pairs are represented by circles. Filled circles denote functionally connected PC to SOM pairs. Open circles are unconnected pairs.

b. There was no change in the relative proportion of different connection types following DE (Chi-squared test, $\chi^2 = 0.39$, p = 0.8235).

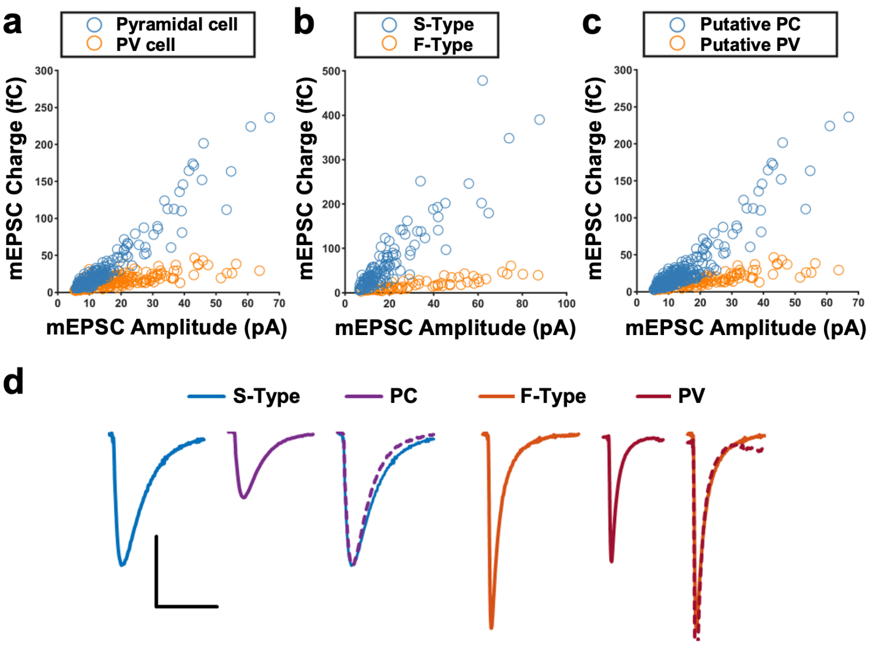

Figure S10. The two types of V1 L2/3 SOM mEPSCs resemble those in V1 L2/3 PCs and parvalbumin cells (PVs)

a. mEPSCs from V1 L2/3 PCs and PVs show distinct kinetics. 200 mEPSCs from a V1 L2/3 PC (blue) and 200 mEPSCs from a V1 L2/3 PV (orange). PC results are reanalysis of mEPSC data from a previously published study [1] and PV data are obtained by recording mEPSCs from PV-Ai14 mice (PV-Cre x Ai14; PV-Cre: Jax stock # 008069, Ai14: Jax stock # 007914).
b. S-Type and F-Type mEPSCs from V1 L2/3 SOMs show similarly distinct kinetics. 200 mEPSCs from a V1 L2/3 SOM.
c. Results from pooling PC and PV mEPSCs from panel a and clustering them with the same method applied to SOM mEPSCs. mEPSCs in the slower "putative PC" clusters are labeled in blue and the faster "putative PV" cluster are labeled in orange.
d. Comparisons of average traces. S-Type (blue) and F-Type (orange) traces are from the NR cells shown in Fig. 1 and Fig. S1. The PC trace (purple) is from 9 V1 L2/3 PCs. The PV trace (red) is from 17 V1 L2/3 PVs. The dashed purple and red traces are scaled PC and PV traces, respectively. Scale bar: 10 pA, 10 ms.

Supplementary Tables

Table S1. Results from linear mixed-effects models fit to connection types for uEPSC recordings shown in Figures 3, 5, and S7.

Strength		AP			Freq			AP:Freq		
		t	p	Power	t	p	Power	t	p	Power
NR	S	0.23	0.8163	–	**2.01**	**0.0453**	**0.63**	**3.18**	**0.0017**	**0.92**
NR	F	**2.08**	**0.0407**	**0.86**	0.46	0.6464	–	1.33	0.1873	–
NR	M	0.77	0.4403	–	0.00	0.9972	–	**4.10**	**<0.0001**	**0.98**
DE	S	0.75	0.4533	–	1.73	0.0847	–	**4.75**	**<0.0001**	**1.00**
DE	F	1.38	0.1692	–	**2.82**	**0.0056**	**0.76**	0.59	0.5564	–
DE	M	0.41	0.6825	–	0.23	0.8174	–	**5.46**	**<0.0001**	**1.00**

Success		AP			Freq			AP:Freq		
		t	p	Power	t	p	Power	t	p	Power
NR	S	1.53	0.1269	–	**2.34**	**0.0201**	**0.73**	1.77	0.0779	–
NR	F	**2.56**	**0.0123**	**0.98**	0.01	0.9880	–	1.29	0.2012	–
NR	M	**4.08**	**<0.0001**	**1.00**	**3.15**	**0.0017**	**0.98**	1.87	0.0618	–
DE	S	0.74	0.4627	–	1.26	0.2084	–	**5.00**	**<0.0001**	**1.00**
DE	F	1.48	0.1418	–	**3.54**	**0.0005**	**0.98**	0.16	0.8739	–
DE	M	1.38	0.1692	–	**2.08**	**0.0385**	**0.66**	**3.76**	**0.0002**	**0.97**

Potency		AP			Freq			AP:Freq		
		t	p	Power	t	p	Power	t	p	Power
NR	S	1.41	0.1608	–	0.30	0.7625	–	**2.21**	**0.0285**	**0.82**
NR	F	0.53	0.5988	–	1.40	0.1663	–	1.73	0.0870	–
NR	M	0.08	0.9328	–	0.55	0.5856	–	**3.62**	**0.0003**	**0.97**
DE	S	0.36	0.7215	–	0.71	0.4808	–	0.99	0.3250	–
DE	F	1.48	0.4121	–	**2.00**	**0.0480**	**0.59**	0.22	0.8288	–
DE	M	0.92	0.3606	–	0.91	0.3619	–	**2.84**	**0.0048**	**0.91**

Async		AP			Freq			AP:Freq		
		t	p	Power	t	p	Power	t	p	Power
NR	S	1.75	0.0823	–	1.89	0.0607	–	**4.49**	**<0.0001**	**1.00**
NR	F	0.80	0.4273	–	0.59	0.5545	–	1.97	0.0516	–
NR	M	**2.43**	**0.0155**	**0.84**	**2.43**	**0.0156**	**0.74**	**7.01**	**<0.0001**	**1.00**
DE	S	0.06	0.9556	–	0.88	0.3819	–	1.15	0.2527	–
DE	F	0.24	0.8078	–	0.42	0.6740	–	**2.37**	**0.0190**	**0.70**
DE	M	**2.15**	**0.0326**	**0.69**	1.26	0.2072	–	**5.46**	**<0.0001**	**1.00**

Linear mixed effects models were described by: *Parameter ~ 1 + AP*Freq + (1|Cell)*. P-values for coefficient t-statistics were calculated using Satterthwaite's method. Post-hoc observed power was calculated using Monte Carlo simulations. Each combination of row and column indicates a separate model. Legend: AP, effect of presynaptic stimulus number; Freq, effect of presynaptic

stimulus frequency; AP:Freq, interaction between effects of AP and Freq; t, t-statistic; p, p-value; Power, post hoc observed power; green entries, model terms considered as significant ($p < 0.05$ and Power > 0.8); yellow entries, model terms that are potentially, but not considered as, significant ($p < 0.05$ and Power < 0.8); unshaded entries, model terms not considered significant ($p \geq 0.05$).

Table S2. Results from linear mixed-effects models fit to all pair data for uEPSC recordings shown in Figures 3, 5, and S7.

All Pair Data	Strength			Success			Potency			Async		
	F	p	Power	F	p	Power	F	p	Power	F	p	Power
AP	0.24	0.6278	–	8.57	0.0035	0.95	1.12	0.2896	–	15.03	0.0001	1.00
Frequency	1.08	0.2994	–	5.30	0.0215	0.69	0.61	0.4349	–	16.63	<0.0001	0.99
Type	4.68	0.0094	0.94	17.25	<0.0001	1	1.05	0.3500	–	6.05	0.0024	0.96
Exp	5.09	0.0242	0.78	0.02	0.8977	–	1.18	0.2766	–	0.42	0.5186	–
Exp:Type	15.17	<0.0001	1.00	17.87	<0.0001	1	17.60	<0.0001	1.00	3.46	0.0318	0.79
Exp:AP	4.86	0.0277	0.71	3.00	0.0837	–	0.5237	0.4694	–	0.0000	0.9971	–
Exp:Frequency	7.26	0.0071	0.81	0.69	0.4055	–	4.55	0.0331	0.75	0.00	0.9799	–
Type:AP	11.69	<0.0001	1.00	2.39	0.0921	–	7.42	0.0006	1.00	6.30	0.0019	0.98
Type:Frequency	18.03	<0.0001	1.00	8.38	0.0002	0.99	5.81	0.0031	0.96	19.99	<0.0001	1.00
AP:Frequency	33.00	<0.0001	1.00	20.53	<0.0001	1	20.16	<0.0001	–	87.59	<0.0001	1.00

Exp:Type Contrasts			Strength			Success			Potency			Async		
			z	p		z	p		z	p		z	p	
NR	F	S	1.48	0.5694		6.93	<0.0001		-2.89	0.0282		–	–	
	F	M	-4.70	<0.0001		-2.86	0.3034		-6.79	<0.0001		–	–	
	M	S	8.53	<0.0001		14.09	<0.0001		4.66	<0.0001		–	–	
DE	F	S	-0.88	0.9180		-1.15	0.7883		-0.06	1.0000		–	–	
	F	M	-1.88	0.3173		-7.59	<0.0001		0.31	0.9992		–	–	
	M	S	0.97	0.8838		6.76	<0.0001		-0.38	0.9979		–	–	
S	DE	NR	2.50	0.0832		5.06	<0.0001		-0.33	0.9990		–	–	
F	DE	NR	-0.36	0.9985		-3.88	0.0009		2.45	0.0935		–	–	
M	DE	NR	-6.47	<0.0001		-2	0.2521		-7.60	<0.0001		–	–	

Top: each column indicates a separate model described by: *Parameter ~ 1 + AP*Freq + AP*Type + AP*Exp + Freq*Type + Freq*Exp + Type*Exp + (1|Cell)*. F-statistics were computed using Type III ANOVA with Satterthwaite's method. Bottom: additional post hoc comparisons were made across levels of Type and Exp for models in which there was a significant interaction between Exp and Type. Z-statistics were computed using z-tests with Dunnett's correction for multiple comparisons. Post-hoc observed power was calculated using Monte Carlo simulations. Legend: AP, effect of presynaptic stimulus number; Freq, effect of presynaptic stimulus frequency; Type, effect of connection type; Exp, effect of experience (NR vs DE); terms with ':', interaction between effects of listed terms; t, t-statistic; p, p-value; Power, post hoc observed power; green entries, model terms considered as significant (top: $p < 0.05$ and Power > 0.8; bottom: $p < 0.05$);

yellow entries, model terms that are potentially, but not considered as, significant ($p < 0.05$ and Power < 0.8); unshaded entries, model terms not considered significant ($p \geq 0.05$).

Table S3. Results from linear mixed-effects models fit to results shown in Figures 4 and S8.

# of APs Elicited	Ideal			Average		
	F	p	Power	F	p	Power
Type	300.91	<0.0001	1.00	329.81	<0.0001	1.00
Exp	74.64	<0.0001	1.00	6.78	0.0116	0.79
Freq	480.60	<0.0001	1.00			
Type:Exp	30.26	<0.0001	1.00	17.90	<0.0001	1.00
Type:Freq	51.74	<0.0001	1.00			
Exp:Freq	12.80	0.0004	0.96			
Type:Exp:Freq	39.53	<0.0001	1.00			

Each column indicates a separate model. Ideal: Model was described by: *Parameter ~ Type*Exp*Freq + (1|Cell)* and was fit to data presented in Figures 4. Average: Model was described by: *Parameter ~ Type*Exp + (1|Cell)* and fit to data presented in Fig. S8. F-statistics were computed using Type III ANOVA with Satterthwaite's method. Post-hoc observed power was calculated using Monte Carlo simulations. Type: Effect of connection type (S-, F-, or Mixed). Exp: Effect of experience (NR or DE). Freq: Effect of frequency of played back uEPSC trains (10 Hz or 40 Hz). Terms with ":" indicate interaction between effects of listed items. Abbreviations: F, F-statistics; p, p-value; Power, post-hoc observed power. Color codes: green, model terms considered as significant ($p < 0.05$ and Power > 0.8); yellow, model terms that are potentially, but not considered as significant ($p < 0.05$ and Power < 0.8).

Table S4. Post-hoc contrasts of estimated marginal means from the model described in Table S3 "Ideal" column (data presented in Figure 4).

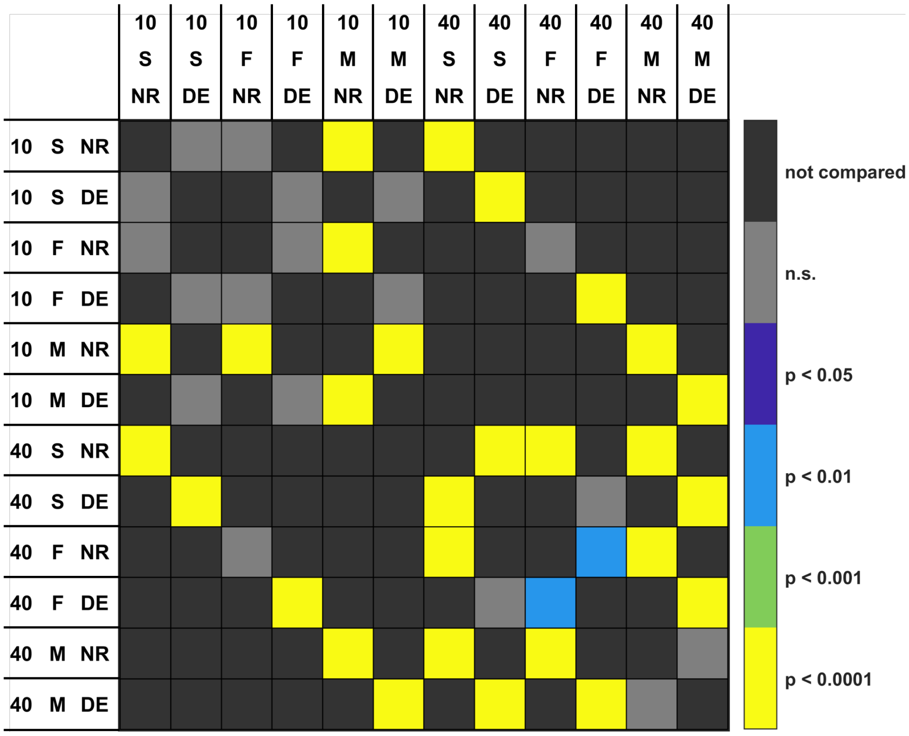

Relevant post-hoc comparisons were made across levels of Exp, Type, and Freq. P-values were computed using z-tests with Dunnett's correction for multiple comparisons. P-values are color coded for each pair as noted in the side bar. Dark gray: comparisons not included in the contrast matrix. Light gray (n.s.): contrasts that were not statistically significant.

Table S5. Post-hoc contrasts of estimated marginal means from the model described in Table S3 “Average” column (data presented in Figure S8).

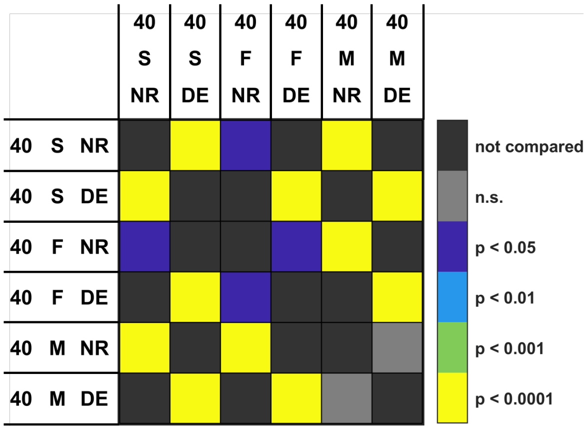

Relevant post-hoc comparisons were made across levels of Exp and Type. P-values were computed using z-tests with Dunnett’s correction for multiple comparisons. P-values are color coded for each pair as noted in the side bar. Dark gray: comparisons not included in the contrast matrix. Light gray (n.s.): contrasts that were not statistically significant.

Supplementary References

1. Chokshi, V., *et al.* Naturalistic Spike Trains Drive State-Dependent Homeostatic Plasticity in Superficial Layers of Visual Cortex. *Front Synaptic Neurosci* **13**, 663282 (2021).
