# Supplementary material for: Selective plasticity of fast and slow excitatory synapses on somatostatin interneurons in adult visual cortex
Source: Nat Commun. 2023 Nov 7;14:7165. doi: 10.1038/s41467-023-42968-y (PMC10630508; doi:10.1038/s41467-023-42968-y)
Supplement: Supplementary file 3 — Reporting Summary [file 41467_2023_42968_MOESM3_ESM.pdf]

nature portfolio

Corresponding author(s): Hey-Kyoung Lee

Last updated by author(s): Oct 11, 2023

Reporting Summary

Nature Portfolio wishes to improve the reproducibility of the work that we publish. This form provides structure for consistency and transparency in reporting. For further information on Nature Portfolio policies, see our Editorial Policies and the Editorial Policy Checklist.

Statistics

For all statistical analyses, confirm that the following items are present in the figure legend, table legend, main text, or Methods section.

n/a	Confirmed	
☐	☒	The exact sample size (*n*) for each experimental group/condition, given as a discrete number and unit of measurement
☐	☒	A statement on whether measurements were taken from distinct samples or whether the same sample was measured repeatedly
☐	☒	The statistical test(s) used AND whether they are one- or two-sided<br>*Only common tests should be described solely by name; describe more complex techniques in the Methods section.*
☐	☒	A description of all covariates tested
☐	☒	A description of any assumptions or corrections, such as tests of normality and adjustment for multiple comparisons
☐	☒	A full description of the statistical parameters including central tendency (e.g. means) or other basic estimates (e.g. regression coefficient) AND variation (e.g. standard deviation) or associated estimates of uncertainty (e.g. confidence intervals)
☐	☒	For null hypothesis testing, the test statistic (e.g. *F*, *t*, *r*) with confidence intervals, effect sizes, degrees of freedom and *P* value noted<br>*Give P values as exact values whenever suitable.*
☒	☐	For Bayesian analysis, information on the choice of priors and Markov chain Monte Carlo settings
☐	☒	For hierarchical and complex designs, identification of the appropriate level for tests and full reporting of outcomes
☒	☐	Estimates of effect sizes (e.g. Cohen's *d*, Pearson's *r*), indicating how they were calculated

Our web collection on statistics for biologists contains articles on many of the points above.

Software and code

Policy information about availability of computer code

Data collection	IGOR Pro v8.04 (Wavemetrics) custom-made macro (publicly available on Github - github.com/heykyounglee). We also uploaded to Mendeley Database (doi: 10.17632/42p6m5638j.2).
Data analysis	IGOR Pro v8.04 (Wavemetrics) custom-made macro (publicly available on Github - github.com/heykyounglee and at Mendeley database 10.17632/42p6m5638j.2) and open-source macro (NeuroMatic v3.0p). MatLab custom-written program (publicly available at github.com/bdgrier).

For manuscripts utilizing custom algorithms or software that are central to the research but not yet described in published literature, software must be made available to editors and reviewers. We strongly encourage code deposition in a community repository (e.g. GitHub). See the Nature Portfolio guidelines for submitting code & software for further information.

Data

Policy information about availability of data

All manuscripts must include a data availability statement. This statement should provide the following information, where applicable:

- Accession codes, unique identifiers, or web links for publicly available datasets
- A description of any restrictions on data availability
- For clinical datasets or third party data, please ensure that the statement adheres to our policy

Data reported in this study are publicly available at Mendeley Data (doi: 10.17632/gpg8jnctrn.1). The hold on the DOI link will be removed when the final version of

the manuscript is submitted for publication. We used the Allen Institute Synaptic Physiology dataset (https://portal.brain-map.org/explore/connectivity/synaptic-physiology): this link is provided in our reference section and referenced in the manuscript.

Research involving human participants, their data, or biological material

Policy information about studies with human participants or human data. See also policy information about sex, gender (identity/presentation), and sexual orientation and race, ethnicity and racism.

Reporting on sex and gender	N/A
Reporting on race, ethnicity, or other socially relevant groupings	N/A
Population characteristics	N/A
Recruitment	N/A
Ethics oversight	N/A

Note that full information on the approval of the study protocol must also be provided in the manuscript.

Field-specific reporting

Please select the one below that is the best fit for your research. If you are not sure, read the appropriate sections before making your selection.

☒ Life sciences ☐ Behavioural & social sciences ☐ Ecological, evolutionary & environmental sciences

For a reference copy of the document with all sections, see nature.com/documents/nr-reporting-summary-flat.pdf

Life sciences study design

All studies must disclose on these points even when the disclosure is negative.

Sample size	Sample size were determined by power analysis of preliminary and prior data sets.
Data exclusions	Recordings from cells that do not meet the inclusion parameters for Rs, Ri, RMS noise, stability of recording, minimum event/trace number (values specified in Methods) were excluded from analysis.
Replication	All results were replicated across several mice of both sexes. The number of replications (i.e. number of mice) are listed under each figure legend and the details of the animal (age, sex) are specified in the source data files.
Randomization	Mice were randomly assigned to each experimental group.
Blinding	The same standardized data analysis pipelines were applied blind to the experimental groups.

Reporting for specific materials, systems and methods

We require information from authors about some types of materials, experimental systems and methods used in many studies. Here, indicate whether each material, system or method listed is relevant to your study. If you are not sure if a list item applies to your research, read the appropriate section before selecting a response.

Materials & experimental systems

n/a	Involved in the study	
☒	☐	Antibodies
☒	☐	Eukaryotic cell lines
☒	☐	Palaeontology and archaeology
☐	☒	Animals and other organisms
☒	☐	Clinical data
☒	☐	Dual use research of concern
☒	☐	Plants

Methods

n/a	Involved in the study	
☒	☐	ChIP-seq
☒	☐	Flow cytometry
☒	☐	MRI-based neuroimaging

Animals and other research organisms

Policy information about studies involving animals; ARRIVE guidelines recommended for reporting animal research, and Sex and Gender in Research

Laboratory animals	Males and female mice: GIN line (TG(GadGFP)4570Swn, Jax stock#003718), PV-Ai14 mice (PV-Cre, Jax stock#008069; Ai14, Jax stock#007914). Ages of the mice used are : P90~113 and P14~16 as noted for each study. Animal room conditions (humidity & temperature) are provided in the Methods.
Wild animals	No wild animals were used.
Reporting on sex	Both sexes were used.
Field-collected samples	No Field-collected samples were used.
Ethics oversight	Johns Hopkins University Animal Care and Use Committee (ACUC).

Note that full information on the approval of the study protocol must also be provided in the manuscript.
